# An *in vitro* study of different material properties of Biodentine compared to ProRoot MTA

**DOI:** 10.1186/s13005-015-0074-9

**Published:** 2015-05-02

**Authors:** Markus Kaup, Edgar Schäfer, Till Dammaschke

**Affiliations:** Department of Operative Dentistry, Westphalian Wilhelms-University, Albert-Schweitzer-Campus 1, building W 30, 48149 Münster, Germany; Central Interdisciplinary Ambulance in the School of Dentistry, Albert-Schweitzer-Campus 1, building W 30, 48149 Münster, Germany

**Keywords:** Biodentine, Radiopacity, Setting time, Solubility, Vickers microhardness

## Abstract

**Introduction:**

The aim of this study was to compare solubility, microhardness, radiopacity, and setting time of Biodentine with ProRoot MTA.

**Methods:**

Solubility in distilled water, radioopacity, and setting time were evaluated in accordance with International Standard ISO 6876:2001. In addition, the solubility in Phosphate Buffered Saline (PBS) buffer was determined. For microhardness-testing, ten samples of each cement were produced. All samples were loaded with a diamond indenter point with a weight of 100 g for 30s.

All data were analysed using the Student-*t*-test.

**Results:**

Both materials fulfilled the requirements of the International Standard ISO 6876:2001 and showed a solubility of <3% after 24 h. At all exposure times Biodentine was significantly more soluble than ProRoot MTA (p < 0.0001). After immersion in PBS-buffer a precipitation of hydroxyapatite was visible.

The Vickers microhardness for Biodentine was significantly higher (62.35 ± 11.55HV) compared with ProRoot MTA (26.93 ± 4.66HV) (p < 0.0001).

ProRoot MTA was significantly more radiopaque (6.40 ± 0.06 mm Al) than Biodentine (1.50 ± 0.10 mm Al) (p < 0.0001).

The setting time for Biodentine (85.66 ± 6.03 min) was significantly lower than for ProRoot MTA (228.33 ± 2.88 min) (p < 0.0001).

**Conclusions:**

Biodentine and ProRoot MTA displayed different material properties. The solubility of both cements was in accordance with the International Standard ISO 6876:2001, whereas ProRoot MTA showed a significantly lower solubility. With regard to microhardness, Biodentine may be used to replace dentine. The radioopacity of Biodentine did not fulfil the requirements laid down in the International Standard ISO 6876:2001. The setting time for ProRoot MTA is significantly higher. Both materials can be used in different indications where specific material properties may be favourable. Hence, the here tested material properties are of clinical relevance.

## Introduction

Mineral Trioxide Aggregate (MTA; Pro Root MTA, Dentsply Tulsa Dental, Tulsa, OK, USA) is a cement which contains different oxide compounds (sodium- and potassium oxides, calcium oxide, silicon oxide, ferric oxide, aluminium oxide and magnesium oxide) and was introduced in dentistry by Torabinejad and White in the mid 1990s. MTA can be denoted as calcium silicate cement, because its composition is similar to a refined Portland cement, which is available in most hardware stores, mixed with bismuth for radioopacity (Table 1) [1,2].

**Table 1:** **Main composition of ProRoot MTA (Dentsply Tulsa Dental, Tulsa, OK, USA) according to [** 63 **]**

**Powder**	**Percentage**
tricalcium silicate (CaO)_3_ **·** SiO_2_	75 wt%
dicalcium silicate (CaO)_2_ **·** SiO_2_	
tricalcium aluminate (CaO)_3_ **·** Al_2_O_3_	
bismuth oxide Bi_2_O_3_	20 wt%
gypsum CaSO_4_ **·** 2 H_2_O	5 wt%
**Liquid**	**Percentage**
distilled water H_2_O	100%

In order to set, it must first be mixed with water. If set MTA gets in contact with tissue fluids, its calcium oxide converts into calcium hydroxide (Ca(OH)_2_). The Ca(OH)_2_-molecule dissociates into calcium and hydroxyl ions, thereby increasing the pH value to approximately 12.5 and resulting in the release of calcium ions [3-5]. MTA can be used as endodontic reparation cement for root-end fillings, apical plug formation, closure of radicular perforations and for direct pulp capping according to the manufacturer’s product information (Dentsply Tulsa Dental, Tulsa, OK, USA). Since its introduction into the market, the material was examined in several studies and is meanwhile well accepted and widely used for this purpose in dentistry (for review see [6-8]).

Even though ProRoot MTA appears to be the preferred material in the above mentioned indications with many positive features, the cement does have several drawbacks: the handling can be difficult, the setting time is long, the use in the visible crown area may lead to tooth discoloration, the compressive and flexural strength is lower than dentine (therefore it should not be used as a restorative base), and it is quite expensive [9-13].

Recently, a new bioactive calcium silicate cement, Biodentine (Septodont, St. Maur-des-Fossés, France), was launched on the dental market denoted as a dentine substitute. Biodentine consists of a powder in a capsule and liquid in a pipette. The powder mainly contains tricalcium and dicalcium silicate, the principal component of Portland cement and MTA, as well as calcium carbonate. Zirconium dioxide serves as contrast medium. The liquid consists of calcium chloride in an aqueous solution with an admixture of polycarboxylate (Table 2). The powder is mixed with the liquid in a capsule in a triturator for 30 seconds. During the setting of the cement calcium hydroxide is formed. The consistency of Biodentine reminds of that of phosphate cement. Comparable to MTA Biodentine can be used for the treatment of root perforations or of the pulp floor, internal and external resorption, apical plug formation, root-end filling, pulp capping and pulpotomy, but also for temporary sealing of cavities, and cervical fillings [12,14]. In comparison to ProRoot MTA until now considerably less research about Biodentine is available. Thus, the aim of the present study was to examine different physicochemical properties (solubility, Vickers microhardness, radioopacity, and setting time) of Biodentine in comparison with ProRoot MTA.

**Table 2:** **Composition of Biodentine (Septodont, St. Maur-des-Fossés, France) according to manufacturer’s specification**

**Powder**	**Purpose**
tricalcium silicate Ca_3_SiO_5_ (>70%)	main core material
dicalcium silicate Ca_2_SiO_4_ (<15%)	second core material
zirconium oxide ZrO_2_ (5%)	radio-opacifier
calcium carbonate CaCO_3_ (>10%)	filler
iron oxides (<1%)	shade
**Liquid**	**Purpose**
water H_2_O	main liquid
calcium chloride CaCl_2_ (>15%)	accelerator
hydrosoluble polymer (polycarboxylate)	water reducing agent

Solubility is an important factor in assessing the suitability of materials to be used as restorative materials in dentistry. Lack of solubility is a desired characteristic for root repair cements [15] because endodontic and restorative materials should provide a long-term seal and avoid leakage from the oral cavity and/or the periapical tissue. Consequently, a low solubility in distilled water as proposed in the Standard of the International Standard Organisation (ISO) 6876:2001 [16] is required. From other studies it is known that calcium silicate cements have the ability to form hydroxyapatite crystals on their surface after contact with phosphate containing body fluid [17-19]. But until now, it is unclear in how far these crystals formations may have an influence on the solubility. Thus, the solubility testing was performed in distilled water as well as in phosphate containing Phosphate Buffered Saline (PBS) buffer.

Vickers microhardness (HV) can be defined as the resistance to plastic deformation of the surface of a material after indentation or penetration. The reported microhardness values for sound dentine are in the range of 60–90 HV [20-22]. It would be optimal if the surface hardness of a calcium silicate cement could reach the same range as dentine.

Root-end filling and endodontic repair materials must be radiopaque in order to be able to evaluate the quality of the filling. It is known that the radiopacity of a 1 mm thick dentine layer is equivalent to that of 1 mm of aluminium [23,24]. Therefore, according to ISO 6876:2001 [16], a radiopacity of 3 mm of aluminium is requested for root canal filling materials. Materials with a radioopacity value lower than 3 mm Al are hardly to distinguish from dentine [25]. Hence, ISO 6876:2001 require a minimal radioopacity equivalent of 3 mm thick aluminium [16].

The presence of moisture is usually required for calcium silicate cements to set [26]. A short setting time is helpful to facilitate a tight seal between e.g. the root canal system and the periodontium, while a long setting time may result in difficulties with maintaining consistency of the mixture [27].

The null hypothesis of this study was that all tested material properties of Biodentine are comparable to ProRoot MTA.

## Methods

### Solubility test

With Biodentine and White ProRoot MTA two endodontic cements indicated for the same purpose were included in this study. Biodentine was obtained from Septodont (St. Maur-des-Fossés, France, LOT 48059) and White ProRoot MTA from Dentsply Maillefer (Ballaigues, Switzerland, LOT 10003596).

The solubility tests followed the methodology laid down in ISO 6876:2001 [16] and were determined of immersion of the samples in double-distilled water. In addition, the solubility was determined in PBS buffer, pH 7.4 (AppliChem, Darmstadt, Germany). The specimens’ change in weight was recorded. For all sample preparation stainless steel ring moulds having a height of 1.6 mm (±0.1 mm) and an internal diameter of 20.0 mm (±0.1 mm) were used. All moulds were cleaned in an ultrasound bath with acetone for 15 min. Thereafter a copper wire was fixed at each mould in order to hang the specimens in a glass dish in such way that the surfaces did not touch and the materials remained undisturbed in the dish. Prior to use all moulds were weighed three times (accuracy ± 0.0001 g) and the mean was calculated.

Both tested materials Biodentine and White ProRoot MTA were mixed according to the manufacturers instructions. The ring moulds were placed on a glass plate and filled to slight excess with the mixed material avoiding air entrapment. All samples were left to set in an incubator (Wärme- und Trockenschrank, Heraeus, Hanau, Germany) at 37°C and 95% relative humidity on a grating for 24 h. Using silicone carbide paper (600 grit) excess material was then trimmed to level the surface of the mould. From each material, 72 samples were prepared for immersion in water and 72 samples for immersion in PBS buffer. In each case the 72 samples were divided into six groups of 12, for immersion in water or in PBS buffer for 1 min, 10 min, 1 h, 24 h, 72 h, and 28 d.

Both materials in their ring moulds were weighed (Sartorius type 1801 MPS, Göttingen, Germany) three times prior to the immersion of the samples. The average reading was recorded. All weight measurements were in grams and recorded to four decimal places.

The samples of each material in its ring mould were immersed in a fresh 160 mL aliquot of liquid at 37°C (±1°C) a time for one day and subsequently in fresh 160 mL aliquots at weekly intervals. The specimens were placed in an airtight dish (7 × 10.5 × 8 cm) with 95% - 100% relative humidity such that both surfaces of each sample were freely accessible to the liquid. There was no agitation of the dish. As controls, 24 empty sample moulds together with the copper wire were immersed in distilled water or PBS buffer, respectively, for 28 days, and any changes in weight were recorded.

After the specified immersion period samples of the two cements were removed from the dish using a pair of tweezers, touching only the metal mould. Samples were washed with 3 mL of double-distilled water and allowed to dry at 37°C in an incubator for 24 h. The specimens were placed on a grating in such way that only the metal moulds touched the grating. Thereafter the samples were weighed three times and the mass of the cements was determined to the nearest 0.0001 g. The difference between the original weight of material and its final weight was recorded to the nearest 0.0001 g. This difference in mass was calculated as a percentage of the original weight of the material, recorded to the nearest 0.001%.

### Vickers microhardness

For measurements of Vickers microhardness (HV), Biodentine and ProRoot MTA were mixed according to the manufacturers’ instructions. Both mixed cements were brought into silicon moulds with a size of 10 mm in length, 5 mm in width and 5 mm in height. The cements were vibrated for 1 min with a vibration intensity of 6000 min^−1^ (KV 36, Wassermann Dental-Maschinen, Hamburg, Germany) to avoid the inclusion of air. Subsequently, the samples were covered with parafilm (“M”-Laboratory Film, American CAN Company, Greenwich, CT, USA) and left to set in an incubator at 37°C and 95% relative humidity for 24 h. Ten samples of each cement were produced. One side of the specimens was then trimmed using silicone carbide paper (600 grit).

For the measurement of the microhardness one polished cement surface of each sample was loaded with a diamond indenter point (Durimet, Wetzlar, Germany) with a weight of 100 g for 30 s to produce a stamp mark in a homogeneous region of the cement surface. The diamond indenter produced one impression with two orthogonal diagonals equal in length which were measured immediately after discharge. The microhardness was calculated as following:

$$ HV=0,102*\frac{F}{A}\approx 0,1891*\frac{F}{d^2} $$

$$ A=\frac{d^2}{2* \sin \frac{136}{2}} $$

where F = load in Newton, 0.1891 = Vickers constant; d = arithmetic mean of the two diagonals, A = impression surface in mm^2^, HV = Vickers hardness.

Each cement sample was measured at five defined points resulting in 50 measurements per cement and a total of 100 measurements.

### Radiopacity

For sample preparation stainless steel ring moulds having an internal diameter of 10.0 mm (±0.1 mm) and a height of 1.0 mm (±0.1 mm) were used according to ISO 6876:2001 [16]. Per cement ten samples were produced and allowed to set for 24 h. From each cement one sample was placed on a dental x-ray film (Kodak Insight Dental Film, Film Speed E, LOT 3110641, Carestream Dental, Rochester, NY, USA) together with an aluminium step wedge (1–9 mm). The x-ray exposures were made using a Sirona Heliodent DS x-ray unit (Bensheim, Germany) with a Sirona tube and a 2.5 mm aluminium filter (Bensheim, Germany) added. The tube voltage was 60 kV and the current 7 mA. The exposure time was 120 ms with a constant source-to-film distance of 21 cm. The films were developed, fixed, and dried in an automatic processor (Dürr-Dental XR 24 Nova, Dürr, Bietigheim-Bissingen, Germany).

The densities were measured with a densitometer (Darklight duo ref, Medset, Hamburg, Germany) with a measuring range D = 0 up to D > 4.5 and accuracy for D < 3 ± 0.01.

### Setting time

The setting time was also evaluated according to ISO 6876:2001 [16]. The following test procedure was applied: because both cements need humidity for proper setting, a setting mould with a diameter of 10 mm and a height of 1 mm was made from dental plaster (Hinrizit, Ernst Hinrichs, Goslar, Germany). Prior to the testing of the setting time the mould was stored at 37°C and 95% humidity for 24 h. Both cements were mixed according to the manufacturers’ instruction and filled into the hard plaster mould. Shortly before the setting time indicated by the manufacturer an indenter point was carefully lowered onto the surface of the cement without exerting any further pressure. The indenter point had a diameter of 2 (±0.1) mm, a flat cylindrical end, a height of 5 mm and a weight of 100 (±5) g.

This testing was repeated every minute until on the cement surface an impression was no longer visible. The time from the end of mixing the cement until that point was recorded. For both cements the experiments were performed on six specimens each.

### Statistical analysis

According to the Kolmogorov-Smirnov-test, all data were distributed normally. Thus, differences between the two cements regarding their solubility, Vickers hardness radiopacity, and setting time were analysed using the Student *t*-test at a level of significance of *p* < 0.05.

## Results

### Solubility in distilled water

There was no change in the weight of empty moulds after immersion in water after 28 days. Both materials fulfilled the requirements of ISO 6876:2001 [16] to be less soluble than 3% after 24 h. In the long run the weight loss after 28 days’ immersion in water of Biodentine was 4.610 (±1.402) % and of ProRoot MTA 1.144 (±0.328) % (Table 3). At all exposure times, Biodentine was significantly more soluble than ProRoot MTA (*p* < 0.0001).

**Table 3:** **Solubility of Biodentine and ProRoot MTA in distilled water**

**Material**	**1 min**		**10 min**		**1 h**		**24 h**		**72 h**		**28 d**	
	**Mean**	**(SD)**	**Mean**	**(SD)**	**Mean**	**(SD)**	**Mean**	**(SD)**	**Mean**	**(SD)**	**Mean**	**(SD)**
Biodentine	0.252	(0.100)	0.999	(0.202)	1.437	(0.426)	2.647	(0.583)	3.700	(0.782)	4.610	(1.402)
ProRoot MTA	0.026	(0.017)	0.247	(0.114)	0.763	(0.235)	0.880	(0.237)	0.940	(0.516)	1.144	(0.328)
*p*-value	<0.0001		<0.0001		<0.0001		<0.0001		<0.0001		<0.0001	

Given are the mean percentages with SD of weight loss for each material and for each immersion period. The differences were significantly different (*p* < 0.0001; *t*-test).

### Solubility in PBS buffer

**Figure 1:**
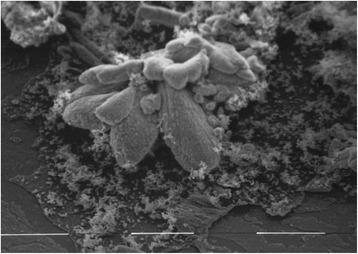
SEM micrograph of the whitish precipitate on the Biodentine surface after 28 d storage in PBS buffer. The form of a lotus flower blossom was described earlier [43]. Original magnification × 6400. The distance between the white bars represents 10 μm.

**Figure 2:**
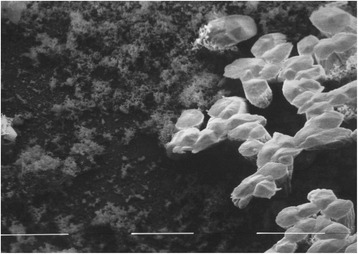
SEM micrograph of the whitish precipitate on the MTA surface after 28 d storage in PBS buffer. The precipitates were more peltiform and thus different from the Biodentine samples. Original magnification × 6400. The distance between the white bars represents 10 μm.

Both cements showed a different solubility in PBS buffer than in distilled water. On the surface of all specimens immersed to PBS buffer longer than 1 h a whitely precipitation was visible in both cements. The deposits adhered on the surfaces even after drying and were hardly to remove. The precipitation of all samples was analysed by Energy Dispersive X-ray Analysis (EDX) and imaged in a scanning electron microscope (SEM) (Figures 1 and 2). (For detailed information about this methodology see [2].) It was found that the precipitation was calcium hydroxyapatite (Ca_10_(PO_4_)_6_(OH)_2_) × n H_2_O).

The solubility of Biodentine in PBS buffer was clearly lower than in distilled water, except the measured value after 24 h. After 28 d even a slight increase in mass could be observed. On the contrary, for ProRoot MTA an increase in mass was detected for all test intervals, except the measured value after 10 min. Thus, it can be concluded that ProRoot MTA is not soluble in PBS buffer. The results for both materials are shown in Table 4.

**Table 4:** **Solubility of Biodentine and ProRoot MTA in PBS buffer**

**Material**	**1 min**		**10 min**		**1 h**		**24 h**		**72 h**		**28 d**	
	**Mean**	**(SD)**	**Mean**	**(SD)**	**Mean**	**(SD)**	**Mean**	**(SD)**	**Mean**	**(SD)**	**Mean**	**(SD)**
Biodentine	0.162	(0.170)	0.253	(0.144)	1.367	(0.264)	3.415	(0.684)	3.274	(1.075)	- 0.053	(0.669)
ProRoot MTA	- 0.029	(0.222)	0.077	(0.074)	- 0.688	(0.098)	- 2.871	(0.256)	- 5.187	(1.019)	- 5.383	(0.501)
*p*-value	<0.0001		<0.0001		<0.0001		<0.0001		< 0.0001		< 0.0001	

Given are the mean percentages with SD of weight loss for each material and for each immersion period. The differences were significantly different (*p* < 0.0001; *t*-test). Negative values mean an increase in weight.

### Vickers microhardness

The mean Vickers microhardness for Biodentine was with 62.35 (±11.55) HV approximately 2.5 fold higher than for ProRoot MTA with 26.93 (±4.66). The differences between Biodentine and ProRoot MTA were highly significant (*p* < 0.0001).

### Radiopacity

ProRoot MTA (6.40 (±0.06) mm Al) was significantly more radiopaque than Biodentine (1.50 (±0.10) mm Al) (*p* < 0.0001). The radiopacity of Biodentine was not in accordance with ISO 6876:2001 [16].

### Setting time

The final setting time was determined to be 85.66 (±6.03) min for Biodentine and 228.33 (±2.88) min for MTA. The difference was statistical highly significant (*p* < 0.0001).

## Discussion

### Solubility in distilled water

The solubility tests performed in the present study followed the methodology of ISO 6876:2001 [16] because Biodentine and ProRoot MTA can be used as root-end filling materials and thereby getting in direct contact with periapical tissue like sealers.

However, while weight loss of the test specimens was recorded by determining the decline in mass of the material samples after storage in water, as already described by some authors [28-31], the International Standard suggests that the increase in weight of the dish in which the samples have been placed (residue method) should be ascertained as the amount of material removed from the specimens [16,32,33]. The specimens were weighed in order to avoid an underestimation of the material going into solution. In order to enhance the accuracy of the measurements, one sample was used for just one immersion period, thus undesirable weight loss of the cements due to repeated drying and immersion was excluded.

It has to be kept in mind that with regard to the strict definition of the physicochemical term solubility, the test used in the present study measured the elution of water-soluble material, but not the solubility. Solubility of a solid is the situation where a pure chemical compound is in thermodynamic equilibrium with its solution [34]. Moreover, it has to be taken into account, that measuring weight differences of the cement specimens may also record disintegration processes that may not be the result of dissolution. For instance, particles of the material may fall out from the cement structure during storage in the liquid [29,34]. Furthermore, water uptake may compensate for dissolved material [29,30,35]. Hence, it can be discussed if a solubility test in distilled water is of clinical relevance. Nevertheless, it was found that both materials fulfilled the requirements of ISO 6876:2001 [16] and showed a solubility of < 3% after 24 h. Biodentine was significant more soluble than ProRoot MTA at all time periods. Under the conditions of this *in vitro* study ProRoot MTA can be described as nearly insoluble. This finding is in accordance with other reports [15,36-38].

On the other hand it must bear in mind that cements like MTA or Biodentine forming calcium hydroxide or calcium oxide during setting should present a certain degree of solubility to improve the mineralization process in contact with vital tissue. OH^−^ and Ca^2+^ release is necessary and related to the solubility of the cements. The alkaline pH and calcium release is linked with the ability to stimulate mineralization. It was shown that materials more soluble than MTA had higher OH^−^ and Ca^2+^ release [39].

### Solubility in PBS buffer

In addition to the solubility test in distilled water, in the present study the solubility of Biodentine and ProRoot MTA was also evaluated in PBS buffer. This was assessed for a better understanding of the advantages of bioactive components to be released from calcium silicate cements [40]. From other studies it is known that these cements have the ability to form hydroxyapatite crystals on their surface after contact with phosphate containing liquids like body fluid or PBS buffer [17-19]. Calcium ions, the dominant ion released from calcium silicate cements, may react with the phosphate in the PBS buffer to hydroxyapatite [17]. In the present study, this could be confirmed for MTA as well as for Biodentine.

The negative values given in Table 4 mean an increase in weight. Thus, it may be concluded that nearly all MTA samples absorbed mass from the PBS buffer. In the Biodentine group this was only observed after 28 d. It may be speculated that Biodentine may release a higher amount of calcium ions in the PBS buffer which may explain the higher solubility in contrast to MTA. In other studies Biodentine showed a higher level of calcium ion release than MTA after storage in Hank’s Balanced Salt Solution (HBSS) and PBS buffer, respectively [41,42]. This may explain the present results.

The SEM micrographs showed the pattern of crystallization of Biodentine, which resembles a lotus flower blossom (Figure 1) as described earlier [43]. In contrast the precipitates found on the MTA samples are more peltiform (Figure 2). The reason for the different crystallization forms is unknown yet. Thus, further research to analyse the exact chemical composition of the white precipitate and to evaluate the increase in weight after storage in PBS buffer is under preparation.

### Vickers microhardness

The measurement of the Vickers microhardness was undertaken with 5 mm thick samples to simulate clinical application. Matt *et al.* recommended 5 mm thick ProRoot MTA as an apical barrier, which was significantly harder than a barrier of 2 mm [44]. In addition the minimal thickness for ProRoot MTA given in the literature as root-end filling material is 3 mm [45] and for apical plug formation 4 mm [46].

Regarding Vickers microhardness, it was found that Biodentine was significantly harder than ProRoot MTA. In the present study, the Vickers microhardness for ProRoot MTA was quite low compared with that derived from other publications where the microhardness of ProRoot MTA was found to be between about 40 HV and 60 HV [36,47-51]. This can be related to different experimental setups.

The Vickers microhardness for Biodentine was about 62 HV after one day which is in accordance with the results of Pradelle-Plasse *et al.* [52] who reported values between 51 HV after 2 h and 69 HV after 1 month.

Camilleri found a Vickers microhardness of even about 130 HV which decreased to about 90 HV after 1 min etching with 35% phosphoric acid [53], whereas in a recent publication a Vickers microhardness of 48.4 HV after 28 d immersion in Hank’s balanced salt solution (HBSS) was reported [54]. These slightly lower values may be related to the storage in HBSS.

Nevertheless, the Vickers microhardness of sound human dentine is about 60 HV and 90 HV [20-22] and thus approximately identical to Biodentine but more than 2-fold higher than that of ProRoot MTA as found in this study. It can be concluded that Biodentine may have a mechanical behaviour similar to human dentine whereas ProRoot MTA seams to be unsuitable for long-term clinical use as a restorative base or to replace dentine. Under this aspect, Biodentine may be used as a dentine substitute.

### Radiopacity

According to the present results, ProRoot MTA was significantly more radiopaque than Biodentine. The radioopacity of ProRoot MTA was found to be 6.5 mm Al, which is in accordance with the current literature [15,36,55-60].

The radioopacity value of Biodentine was found to be 1.5 mm Al thickness. This is in contrast to the manufacturer’s information who claimed that Biodentine possess a radiopacity of 3.5 mm Al and also in contract to the results of a study by Grech *et al.* in which Biodentine displayed a radiopacity of 3.3 to 4.1 mm Al after immersion in HBSS for 1 d and 28 d, respectively [54]. In a recent study values of 2.8 ± 0.48 mm Al were reported [61]. Thus, the wide range of radiopacities values of Biodentine as given so far in the literature (2.8 to 4.1 mm Al) together with the finding of the present study may indicate a certain lack of product standardisation. Certainly, additional investigation are warranted to further elucidate the discrepancy in the values given by the manufacturer or by other authors [54,61] and the results of the present study, yet. However, in contrast to ProRoot MTA Biodentine is hardly to distinguish from dentine radiographically, which may support the present results.

The difference in radiopacity between Biodentine and ProRoot MTA may be explained by the use of different radiopacifieres. Whereas ProRoot MTA contains about 2 at% bismuth [1,2], 5% of zirconium oxide (ZrO_2_) is added to Biodentine as radiopacifier (according to manufacturer’s information). Under the aspect of biocompatibility zirconium oxide seems to be superior compared to bismuth oxide [60], but obviously the amount seems to be insufficient in Biodentine. A radiopacity value of about 1.5 mm Al for Biodentine is too low for clinical use and should thus be improved.

### Setting time

The initial setting time according to the manufacturer of Biodentine is about 12 min. In the present study, the final setting time of Biodentine was 85.66 (±6.03) min and thus sevenfold longer. Grech *et al.* evaluated the setting time of Biodentine to be 45 min according to ISO 9917–1:2007 [54]. The difference in the determined setting time may be explained by the different ISO standards used. Nevertheless, in both studies the setting time for Biodentine was significantly longer than the initial setting time given in the instructions of the manufacturer and should be taken into consideration when using this cement.

In the present study, the setting time of MTA was 228.33 (±2.88) min, whereas other studies reported setting times for ProRoot MTA between 165 (±5) min [15] and 170 (±2) min [62]. Nevertheless, the setting time for ProRoot MTA was significant longer than for Biodentine, which may be a disadvantage in clinical practice.

## Conclusion

The null hypothesis of this study has to be rejected as Biodentine and ProRoot MTA showed marked differences in their physicochemical properties. Concerning solubility, both materials fulfilled the requirements of ISO 6876:2001 [16] but Biodentine was significantly more soluble than ProRoot MTA. The increased solubility may be an advantage in regard to bioactivity, but further research is necessary. Biodentine possess a significantly higher Vickers microhardness compared to ProRoot MTA. Hence, concerning surface hardness this cement may be denoted as a dentine substitute. The radioopacity value of Biodentine was significantly lower than that of ProRoot MTA and not in accordance with ISO 6876 [16]. The radiodensity of Biodentine should be improved. The setting time of Biodentine is significantly lower than that of ProRoot MTA, which may be an advantage in clinical practice.

## Abbreviations

Ca(OH)_2_: Calcium hydroxide
EDX: Energy Dispersive X-ray Analysis
HBSS: Hank’s balanced salt solution
HV: Vickers microhardness
ISO: International standard organisation
MTA: Mineral trioxide aggregate
PBS: Phosphate buffered saline
SEM: Scanning electron microscope
